# The estimation of healthcare cost of kidney transplantation in Japan using large-scale administrative databases

**DOI:** 10.1007/s10157-024-02551-1

**Published:** 2024-11-20

**Authors:** Masataka Hasegawa, Hirotaka Kato, Takashi Yoshioka, Rei Goto

**Affiliations:** 1 Health Technology Assessment Unit, Department of Preventive Medicine and Public Health, Keio University School of Medicine, 35 Shinano-machi, Shinjuku-ku, Tokyo, 160-8582 Japan; 2 Graduate School of Health Management, Keio University, Tokyo, Japan; 3 School of Economics and Business Administration, Yokohama City University, Yokohama, Japan; 4 Institute of Clinical Epidemiology, Showa University, Tokyo, Japan; 5 Graduate School of Business Administration, Keio University, Tokyo, Japan

**Keywords:** Kidney transplantation, Cost analysis, Large-scale administrative databases

## Abstract

**Background:**

The financial burden of kidney replacement therapy (KRT) is considerable, and detailed information on KRT costs is essential for managing these huge healthcare costs. However, cost analyses for kidney transplantation (KTx) are limited in Japan. This study aimed to report the healthcare costs of KTx recipients in Japan based on large medical receipt data.

**Methods:**

This cost analysis of KTx recipients using the Japan Medical Data Center Claims Database between January 2005 and August 2020 identified living donor KTx (LDKT) and deceased donor KTx (DKT) recipients. The primary outcome was the total direct healthcare costs of KTx recipients. As an exploratory analysis, we examined the factors that contributed to the increase in the costs of LDKT.

**Results:**

In total, 84 LDKT and 17 DKT recipients were included in this study. The total ﻿healthcare costs for LDKT and DKT recipients during the first year after KTx were 6,639,982 and 6,840,450 JPY/year, respectively. However, after the second year post-KTx, total ﻿healthcare costs decreased to 1,735,931 and 1,348,642 JPY/year for LDKT and DKT recipients, respectively. During the first year, inpatient costs accounted for > 70% of the total ﻿healthcare costs, whereas pharmaceutical costs accounted for more than half after the second year post-KTx. The use of everolimus and male sex were associated with higher and lower total healthcare costs in the first and subsequent years after LDKT, respectively.

**Conclusion:**

Using large-scale administrative databases, this study revealed the total ﻿healthcare costs of KTx in Japan and provided valuable information for the health technology assessment of KTx.

**Supplementary Information:**

The online version contains supplementary material available at 10.1007/s10157-024-02551-1.

## Introduction

Kidney failure with replacement therapy (KFRT) is a major global public health issue that affects individuals, healthcare systems, and societies [1]. Patients with KFRT require kidney replacement therapy (KRT), such as dialysis or kidney transplantation (KTx), to sustain their lives. However, KRT consumes substantial healthcare resources, and the treatment duration is usually long; e.g., 7.37 years on average in Japan [2]. Thus, the healthcare cost of KRT is extremely high [3]. In Japan, the total healthcare cost of hemodialysis (HD) is approximately JPY 1.5 trillion, accounting for approximately 3% of the total national healthcare expenditure of JPY 45 trillion in 2021 [4]. However, the cost data for each KRT modality in Japan are scarce. To the best of our knowledge, only one study has reported the cost estimation of HD using claims data. Policymakers should effectively manage the high healthcare costs associated with KFRT and KRT costs. Therefore, detailed information on KRT costs is crucial for policymakers to effectively manage the huge healthcare costs associated with a growing population at risk for KFRT [5] and to provide efficient care for them.

Among KRT, KTx is the preferred modality for patients with KFRT because it improves their survival and quality of life compared with dialysis [6, 7]. Although the cost of KRT varies with treatment modality, the cost of KTx was reported to be 21−88% lower than that of dialysis [8]. For example, the annual costs of dialysis and KTx in the United Kingdom (U.K.) were $29,665 and $21,534, respectively, whereas those in the United States (U.S.) were $145,215 and $80,873, respectively. These annual costs of KTx in U.S. and U.K. were estimated by combining averaging costs from the year of KTx and subsequent years. Furthermore, KTx is more cost-effective compared with other KRT modalities [9, 10].

Japan has the sixth highest incidence (307 per million people) and third highest prevalence (2749 per million people) of KFRT [11]. Given the severity of health and economic finances in Japan and the burden of KFRT on society, it is critical to further discuss the health and economic aspects of KRT modalities. However, cost analysis of KTx is significantly limited in Japan. To the best of our knowledge, only two studies have reported the cost of KTx in Japan. One study reported that the cost of KTx in Japan was roughly 6.7 million JPY for living donor KTx (LDKT) and approximately 8.5 million JPY for deceased donor KTx (DKT) [12]. Another study reported that the costs of ABO-compatible and ABO-incompatible LDKT were approximately 6.6 and 7.8 million JPY, respectively [13]. Notably, both studies were based on retrospective data collected from a single university hospital. Former cost data have been repeatedly used in cost-effectiveness research in Japan [14]; however, the study results may not accurately represent the situation of the entire country because they were collected from a single institution. Healthcare costs for individual diseases exhibit inter-hospital variability, attributed to differences in clinical management approaches and patient demographics. This variation has also been reported in Japanese acute-hospital settings [15, 16]. Given the variation in cost among institutions, the cost estimation derived from a single institution did not represent the comprehensive cost profile of Japanese KTx, and cost-effectiveness analyses based on this research may not consider the uncertainty of cost parameters, leading to the risk of incorrect decision-making in therapy or policy. Therefore, estimating the cost analysis of KTx at the national level has become an emerging issue to ensure accurate cost data and provide information for the health technology assessment of KRT and KTx in Japan.

This study aimed to estimate the healthcare costs associated with KTx in Japan. To the best of our knowledge, this is the first study to report healthcare KTx costs in Japan using an analysis of large medical receipt data.

## Methods

### Study design and data sources

This retrospective observational study used large-scale administrative databases derived from Japan Medical Data Center (JMDC), Inc. (https://www.jmdc.co.jp, Tokyo, Japan) to evaluate the costs and treatment patterns of patients who underwent KTx in Japan. The JMDC database covers epidemiological and medical examination data from > 1300 insurers and approximately 17 million beneficiaries from 2005 (as of May 2024), most of whom are employees of Japanese companies and their family members aged < 74 years. In the JMDC database, every patient is allocated a unique personal identification number to anonymize and prevent double-counted claims, thereby allowing patients to be traced throughout different facilities. Diagnoses are recorded using the International Classification of Diseases, 10th Revision (ICD-10) codes. The study index period was from January 1, 2005, to August 31 2020, to allow at least 1 year of follow-up. The index date was defined as the date of the first KTx. Patients who underwent KTx during the index period were followed-up as much as possible.

### Inclusion and exclusion criteria

Patients who underwent any KTx procedure during the index period were eligible for inclusion. KTx procedures were identified by confirming both the post-transplant diagnosis (ICD-10 code: Z94.0) and Japanese procedure codes corresponding to either the LDKT (K780-2) or DKT (K780) procedure. We imposed the following three exclusion criteria: (1) patients whose claims were not confirmed for at least 12 months after the index month in the database, (2) patients whose annual drug cost estimates were confirmed to be inaccurate (it was determined that the total healthcare cost estimation for patients was inaccurate when their annual drug cost estimation was zero even for 1 year among their annual drug cost estimations for each year), and (3) patients whose cost estimation in the index month was not recorded and those whose cost estimation was recorded after the next month. Given the nature of the JMDC database, it is probable that this phenomenon was a consequence of changes in the health insurance society. Cost estimation in the index month is of utmost importance because it includes the cost of KTx. Consequently, it was determined that the cost estimation for these patients was inaccurate.

### Outcomes variables

The primary outcome was the total direct ﻿healthcare costs per year. These costs included outpatient care, hospitalization care, and pharmaceuticals. It did not include the costs of long-term care or indirect costs such as the productivity loss of patients and their family members. The scope of costs was determined from the perspective of public healthcare payers [17]. In Japan, total direct ﻿healthcare costs are the most important cost information from the perspective of public healthcare payers [18]. The total ﻿healthcare costs were estimated solely for KTx recipients, excluding the costs associated with kidney donation. These costs were estimated annually from the index month until the point at which records could not be observed, when the patient died, or when the patient initiated maintenance dialysis (Japanese procedure codes: J038 or C102) during the index period, indicating that the patient’s graft was lost. To estimate the exact total annual healthcare cost, the costs in the final year for each patient were excluded because cost observation was frequently censored in the middle of the year. The Japanese procedure codes used in this study are provided in Online Resource 1.

### Statistical analyses

Our main analyses included descriptive analyses of the characteristics and healthcare costs of the LDKT and DKT recipients. The values closest to the index date during the baseline period were used as baseline characteristics. Annual direct ﻿healthcare costs were assessed in terms of hospitalization and outpatient visits, and drug-related costs were also specifically assessed. As an exploratory analysis, we constructed linear regression models to examine the association between total ﻿healthcare costs in the first year after LDKT and the covariates. Linear mixed-effects models were used to explore the influence of different variables on the total ﻿healthcare costs of LDKT recipients in subsequent years. In linear mixed-effects models, patients were treated as random-effects variables. Only patients undergoing LDKT were included in the exploratory analysis because the number of patients undergoing DKT in the JMDC database was expected to be small owing to the small number of DKT recipients throughout Japan. Furthermore, we performed a post-hoc exploratory analysis using age as categorical variable as we observed non-linear relationships in the total ﻿healthcare costs of both the first year of KTx and subsequent years. A detailed explanation of the covariates is provided in Online Resource 2. The costs were presented in JPY (1 USD = 141.6 JPY, as average exchange rate for 2023). Descriptive analyses included numbers and proportions (%) summarized with continuous numerical values characterized by means (standard deviations) or medians (interquartile ranges), as appropriate. Statistical significance was set at *p* < 0.05. All analyses were performed using the STATA software (version 17.0; Stata Corp).

### Ethical considerations

This study used anonymized data from an existing claims database, and the requirements for informed consent and ethical and institutional review board approval were waived.

## Results

### Baseline characteristics

We identified 159 KTx recipients diagnosed with post-transplant conditions (ICD-10 code: Z94.0) who underwent KTx between January 1, 2005, and August 31, 2020. The final analysis included 84 LDKT and 17 DKT recipients from 11 and 13 institutions, respectively (Online Resource 3). Table 1 outlines the recipients’ characteristics. The mean age at transplantation for LDKT recipients was 44.7 ± 1.4 years, with 64.3% of patients being male. In contrast, the mean age at transplantation for DKT recipients was 46.8 ± 3.5 years, with 65% of patients being male. The proportion of preemptive LDKT recipients was 46%.

**Table 1:** Patient characteristics

	Living donor kidney transplant (*n* = 84)	Deceased donor kidney transplant (*n* = 17)
	*n* (%)	*n* (%)
Male	54 (64)	11 (65)
Age at transplantation (years, mean ±SD)	44.7 ± 1.4	46.8 ± 3.5
Cause of KFRT		
DKD	10 (12)	2 (12)
CGN	26 (31)	4 (24)
PKD	16 (19)	5 (29)
Pre-emptive KTx	39 (46)	N/A
ABO-incompatible KTx	17 (20)	N/A
Use of an immunosuppressive drug		
MMF	75 (82)	17 (100)
Everolimus	16 (19)	4 (24)
Tacrolimus	78 (93)	13 (77)
Cyclosporine	7 (8)	5 (29)

Data are shown as counts and frequencies (%) and means ± standard deviations, as appropriate

*KFRT* kidney failure with replacement therapy, *DKD* diabetic kidney disease, *CGN* chronic glomerulonephritis, *PKD* polycystic kidney disease, *KTx* kidney transplantation, *MMF* mycophenolate mofetil, *N/A* not available

### Healthcare costs

**Fig. 1:**
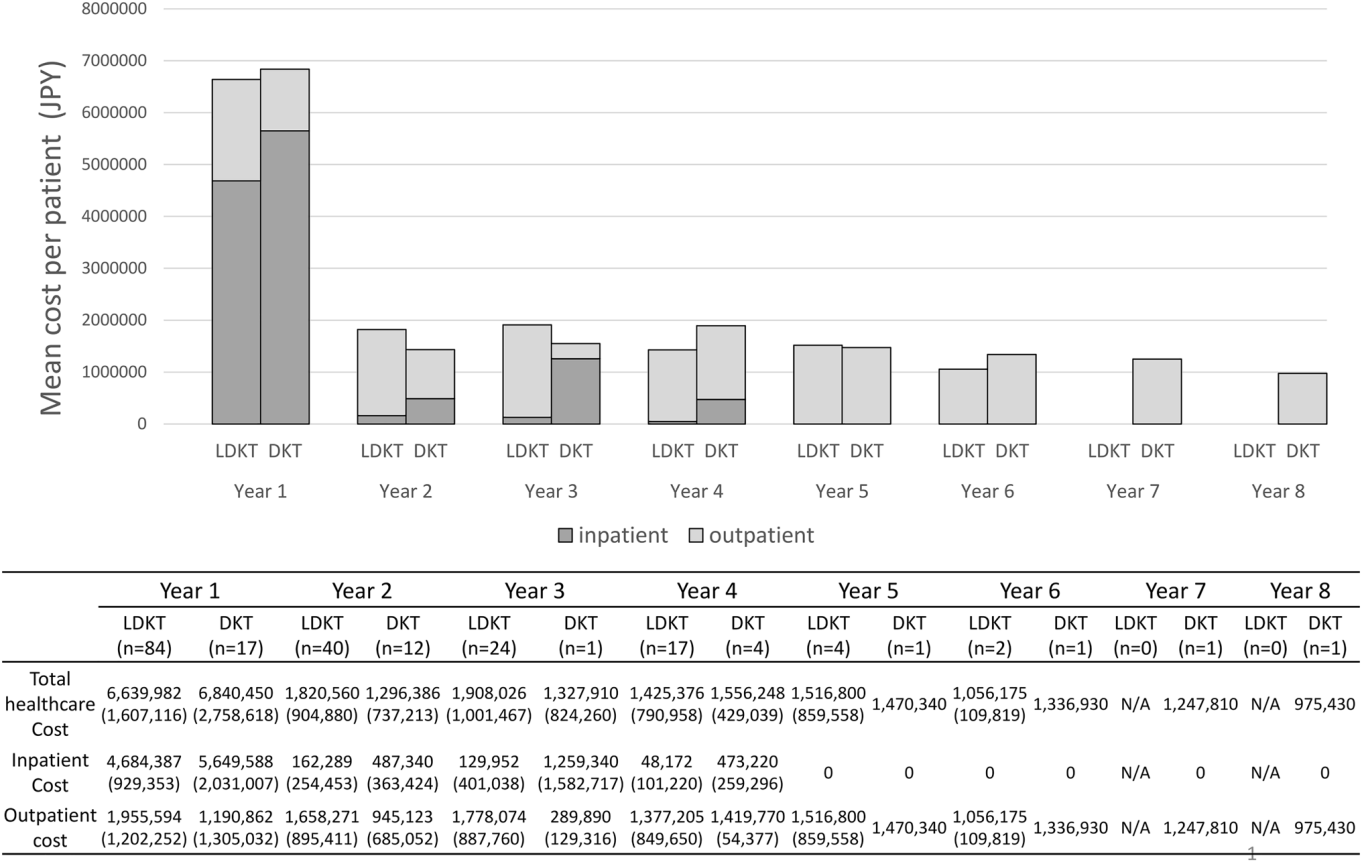
Total direct ﻿healthcare cost of kidney transplantation per patient and by year of follow-up. Data presented in the table are means (standard deviations). *LDKT* living donor kidney transplantation, *DKT* deceased donor kidney transplantation; *N/A* not available

**Fig. 2:**
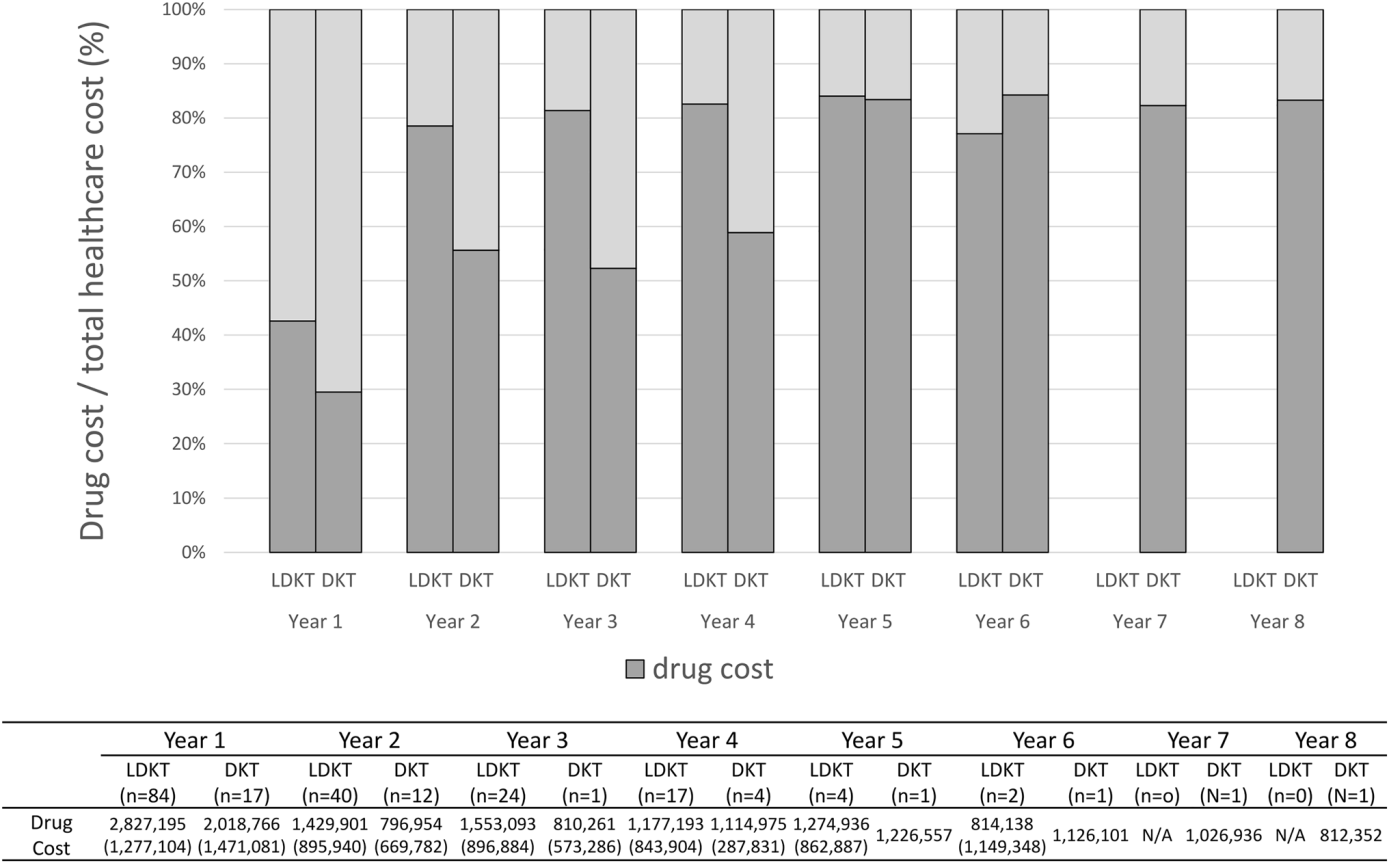
The proportion of the drug cost of kidney transplantation in the total ﻿healthcare medical cost per patient and by year of follow-up. Data presented in the table are means (standard deviations). *LDKT* living donor kidney transplantation, *DKT* deceased donor kidney transplantation, *N/A* not available

The total ﻿healthcare costs [95% confidence interval (CI)] were the highest during the initial year after KTx, averaging 6,639,982 [6,291,216 to 6,988,747] JPY/year and 6,840,450 [5,422,100 to 8,258,800] JPY/year for LDKT and DKT recipients, respectively (Fig. 1). During the first year, inpatient costs accounted for > 70% of the total ﻿healthcare costs, averaging 4,684,387 [4,482,706 to 4,886,069] JPY/year and 5,649,588 [4,605,341 to 6,693,836] JPY/year for LDKT and DKT recipients, respectively. However, from the second year post-KTx, total ﻿healthcare costs decreased and remained relatively stable, at < 2 million JPY/year. Specifically, the average annual costs after the second year post-KTx were 1,735,931 [1,540,067 to 1,931,194] JPY/year for LDKT recipients and 1,348,642 [1,143,488 to 1,553,796] JPY/year for DKT recipients. The result of age-stratified total ﻿healthcare costs for LDKT recipients is provided in the Online Resource 4. The proportion of drug-related costs was less than half of the total ﻿healthcare costs only in the initial year; after the second year following KTx, it accounted for more than half of the total medical costs for LDKT and DKT recipients (Fig. 2). The costs for LDKT recipients associated with patient characteristics are summarized in Table 2.

**Table 2:** Total ﻿healthcare costs of living donor kidney transplantation associated with patient characteristics

	Cost during first year after KTx (JPY)	Cost after second year post-KTx (JPY)
	Mean (SD)	Mean (SD)
Sex		
Male	6,572,583 (1,709,674)	1,526,044 (880,968)
Female	6,761,299 (1,423,560)	2,079,382 (880,011)
Cause of KFRT		
Diabetes	6,572,262 (1,575,628)	851,970 [352,180 to 2,846,290]
Non-diabetes	6,649,133 (1,917,526)	1,837,710 [1,252,750 to 2,177,260]
Pre-transplantation treatment		
Pre-emptive KTx	6,546,902 (1,572,437)	1,719,752 (745,887)
Dialysis	6,720,052 (1,649,974)	1,744,020 (996,462)
ABO-compatible/ABO-incompatible KTx		
ABO-incompatible KTx	6,986,839 (1,406,865)	1,896,260 [1,252,750 to 3,030,100]
ABO-compatible KTx	6,551,973 (1,652,096)	1,788,585 [854,010 to 2,188,790]
Use of everolimus		
Use of everolimus	7,500,369 (2,022,952)	1,718,391 (1,146,231)
Nonuse of everolimus	6,437,538 (1,437,027)	1,739,883 (865,801)

Data are shown as mean ± standard deviation or median [interquartile range], as appropriate

*KTx* kidney transplantation, *JPY* Japanese Yen, *KFRT* kidney failure with replacement therapy

### Exploratory analysis

Table 3 presents the results of exploratory analyses. Regarding the total ﻿healthcare costs of the first year following LDKT, the use of everolimus use was associated with high total cost (1,218,695JPY/year; 95% CI 347,011 to 2,090,379; *p* = 0.007) compared with non-use. However, during a subsequent year after LDKT, male sex was associated with low total ﻿healthcare costs (− 54,137.2 JPY/year; 95% CI − 1,038,727 to − 44,018.0; *p* = 0.034). In the post hoc exploratory analysis (Online Resource 5), recipients aged under 29 years were associated with high total cost (1,160,700 JPY/year; 95% CI 28,497 to 2,292,902; *p* = 0.002) compared with those aged 40 to 49 years for the first year following LDKT.

**Table 3:** Multivariate regression analysis of total medical costs of living donor kidney transplantation

	First year after living donor KTx^a^			A subsequent year after living donor KTx^b^		
	Cost (JPY)	95% CI	*p* value	Cost (JPY)	95% CI	*p* value
Male (ref: female)	− 547,394	− 774,390 to 664,911	0.880	− **54,137.2**	− **1,038,727 to − 44,018.0**	**0.034**
Age at transplantation (continuous per 1 year)	− 26,915	− 53,668 to 838	0.057	− 8893.3	− 36,584.7 to 18,798.0	0.52
ABO-incompatible KTx (ref: ABO-compatible KTx)	747,746	− 115,048 to 161,054	0.088	495,908.2	− 408,285 to 1,400,101	0.27
Presence of DKD (ref: absence of DKD)	− 60,388	− 1,131,110 to 1,010,334	0.911	101,774.2	− 1,187,861 to 1,391,410	0.87
Use of everolimus (ref: non-use of everolimus)	**1,218,695**	**347,011 to 2,090,379**	**0.007**	− 7821.6	− 679,176.8 to 663,533.7	0.98

Bold values are statistically significance

*DKD* diabetic kidney disease, *KTx* kidney transplantation, *JPY* Japanese Yen *CI* confidence interval

^a^Linear regression model

^b^Linear mixed-effect models: In these models, patients were treated as random-effect variables

## Discussion

This study aimed to estimate the healthcare costs of KTx recipients in Japan using large-scale administrative databases. The results showed that the total healthcare costs for LDKT and DKT recipients were approximately 6.6 million JPY/year and 6.8 million JPY/year, respectively. In addition, this study found that the total healthcare costs for both KTx recipients were the highest during the first year after KTx and decreased after the second year following KTx. Inpatient costs accounted for > 70% of total healthcare costs during the first year. However, after the second year post-KTx, pharmaceutical costs accounted for more than half of the total healthcare costs. Exploratory analysis revealed that the use of everolimus was associated with a higher total cost in the first year following LDKT, whereas male sex was associated with a lower total cost in the subsequent year following LDKT.

In Japan, approximately 350,000 individuals received dialysis in 2022, with HD accounting for 98% of KRT [2]. Despite the significant benefits of KTx, only approximately 2% of patients were treated with KTx. Moreover, LDKT accounts for approximately 90% of KTx cases in Japan, and DKT is rarely performed [19]. This is partially attributable to cultural reasons and societal and ethical barriers to organ donation. Needless to say, improving health-related outcomes and the quality of life is of paramount importance. However, it is also crucial to consider and compare the costs of different KRT modalities and understand the costs of transplantation in each country. Numerous studies have been conducted to estimate KTx costs. According to a systematic review, the estimated cost of KTx in the U.S ranges from $34,343 to $80,876, whereas that in the U.K ranges from $14,067 to $31,115 [8]. The variation in estimated KTx costs between the U.S and U.K was attributed to the difference in healthcare systems, cost of care, and clinical practices between countries [20]. Consequently, it is important to estimate the KTx costs in each country to reflect its healthcare circumstances. Nevertheless, in Japan, only two studies have reported the cost of KTx using retrospective data from a single university hospital. One study reported that the estimated costs of LDKT and DKT were approximately 6.7 million JPY/year and 8.5 million JPY/year, respectively, during the first year [12], and this study had a limited sample size, with only 40 LDKT patients and 5 DKT patients. Another study reported that the healthcare costs of ABO-compatible and ABO-incompatible LDKT were approximately 6.6 and 7.8 million JPY during the first year, respectively [13]. Furthermore, this report showed that the cost of ABO-compatible and ABO-incompatible LDKT during the second year was approximately 2.5 million JPY. However, the study period was relatively short: only 2 years after KTx. The cost of medical care for each disease considerably varies among hospitals. Some reports have indicated that there are variations in healthcare costs for each disease in Japanese acute-hospital settings [15, 16]. Generally, clinical practice patterns in major teaching hospitals tend to be more resource-intensive and costly. This trend is partially attributable to consistently longer lengths of stay across all risk levels, as well as higher resource utilization for high-risk patients compared to non-teaching hospitals [21–23]. Although the estimated cost of LDKT was comparable between our study and previous reports, the estimated costs of DKT and ABO-incompatible LDKT in our study were lower than those in an existing study. In our study, 17 DKT recipients were identified from 13 distinct institutions, suggesting that non-university hospitals may also be represented in our sample. Consequently, although our study is subject to selection bias due to the characteristics of the JMDC database, the inclusion of a more diverse range of hospitals may account for the inconsistency of the costs in DKT recipient between our study and the previous report. A similar factor may account for the inconsistency observed in the costs of ABO-incompatible LDKT in our study and another previous report. While 17 ABO-incompatible LDKT recipients were identified from 5 institutions and their average age was 48.2 years in our study, the average age of those in this previous report was 52.1 years. In addition, the higher risk associated with ABO-incompatible KTx often necessitates more intensive immunosuppressive therapy, which may have contributed to the higher costs in the previous report, as higher risk patients are likely to receive KTx at a university hospital. This difference in patient demographics and risk profile could potentially contribute to the observed cost inconsistency, as older and higher risk patients may require more intensive care or have a higher risk of complications. Given the differences in medical practices among institutions, it is difficult to generalize the data from a single university hospital as representative of the estimated cost of KTx in Japan. In our study, LDKT and DKT recipients were identified from 11 and 13 distinct institutions, respectively. The lower number of institutions for LDKT compared to DKT is due to the inclusion of a high-volume center for LDKT and the selection criteria for DKT recipients in Japan. Thus, our study presents more comprehensive KTx cost data in Japan, as it is based on large-scale databases from multiple hospitals that include a more diverse range of patients and medical practice patterns. To the best of our knowledge, this is the first study to estimate the KTx costs in Japan using large-scale administrative databases.

Our exploratory analysis revealed that everolimus use was associated with high total costs in the first year after LDKT. Immunosuppressive drugs are the primary medications for patients with KTx. For patients with a low immunological risk of KTx, maintenance regimens based on calcineurin inhibitors (CNIs) and mycophenolate are effective and safe [21]. Despite the use of mammalian targets of rapamycin inhibitors, including everolimus, CNIs and mycophenolate have remained the most commonly used immunosuppressive agents in KTx for the last 2 decades in various combinations and regimens. Regarding efficacy and safety, an everolimus-based regimen is non-inferior to the standard-of-care regimen of mycophenolate and CNIs in terms of mortality, biopsy-proven acute rejection, and risk of adverse infection [24, 25]. Therefore, our study provides additional information from a healthcare finance perspective when selecting an immunosuppressive regimen. Furthermore, our study also demonstrated that male sex was associated with lower total ﻿healthcare costs during the subsequent years after LDKT. The differences in KTx outcomes between the sexes remain unclear and controversial [26]. Previous research has shown that the risk of kidney allograft failure is generally higher or equal in women compared to men, with the exception of women aged 45 years and older [27, 28]. In our study, the average age of LDKT recipients was 44.6 years. This finding suggests that age may influence not only KTx outcomes but also associated costs. Moreover, other studies have reported that females exhibit lower tacrolimus bioavailability compared to males [29]. This pharmacokinetic difference potentially leads to higher pharmaceutical costs in subsequent years for female LDKT recipients, as they may require higher doses to maintain therapeutic levels. Our findings suggest that the lower costs observed in male recipients could be attributed to a combination of these elements. However, the precise mechanisms underlying these sex-based cost differences remain unclear and require further investigation. We performed a post hoc exploratory analysis using age as categorical variable as we observed non-linear relationships in the total ﻿healthcare costs of both the first year of LDKT and subsequent years. This post hoc exploratory analysis revealed that recipients aged under 29 years were associated with high total costs in the first year after LDKT, and non-linear relationships were observed for age-stratified total ﻿healthcare costs for LDKT recipients. These age-related cost variations may be attributable to different factors across age groups. Generally, costs for younger patients, particularly pediatric recipients, tend to be higher due to more complex surgical procedures and the need for more intensive care. Conversely, costs for older patients may increase due to the management of comorbidities. Thus, the relationship between age and KTx costs was non-linear likely attributable to these age-related cost variations. It is important to note that in our study, the costs for LDKT recipients aged over 60 years were not significantly higher. However, this finding may be due to the small number of recipients in this age group, a limitation inherent to the nature of the JMDC database used in our analysis.

Our study has some limitations. First, the JMDC database is composed of employees of Japanese companies and their family members aged < 74 years, which may have introduced a sampling bias in the sample characteristics. Notably, our study showed a higher proportion of preemptive LDKT recipients (46%) compared to national statistics (28.9%). This inconsistency can be largely attributed to two high-volume centers in our sample. Furthermore, the average age of LDKT recipients at transplantation in our study (44.7 ± 1.4 years) was slightly younger than reported in national epidemiological data (48.9 ± 14.9 years). This age difference likely reflected the demographic composition of the JMDC database. These inconsistencies may introduce a selection bias toward better overall health status in our study population. Consequently, this bias could potentially result in an underestimation of healthcare costs in our analysis. Therefore, it may be difficult to generalize our results to all patients who underwent KTx in Japan. However, it is unlikely that the results of this study would significantly change because there would be few differences between the characteristics of all patients undergoing KTx in Japan and those of the sample from the database because patients who undergo KTx must be in relatively good condition. Furthermore, the age limitation is not a concern, as KTx is recommended to be typically performed on patients aged < 70 years according to the Japanese KTx guideline. Second, the sample size of patients who underwent KTx was small. Owing to the relatively low number of KTx performed in Japan, approximately only 2000 patients undergo KTx annually, and LDKT accounts for approximately 90% of all KTx procedures in Japan. Consequently, the number of cases extracted from the database is inevitably small. In our study, LDKT accounted for 80% of the cases, whereas DKT accounted for only 20%. This discrepancy in the proportions observed between our study and Japanese statistics is likely owing to the nature of the JMDC database. This is because LDKT can also be performed even for patients aged ≥ 70 years in Japan, although the number of patients is limited, if their medical condition is good. However, Japanese KTx statistics from 2022 indicate that the recipient average age and proportion of males were 48.9 years and 63.5% in LDKT and 48.0 years and 64.1% in DKT, respectively [19]. The recipients’ average age and proportion of males in our study were highly comparable to Japanese transplantation statistics. Consequently, despite the limited sample size of patients in our study, the potential impact of selection bias is likely to be small. Prior studies estimating KTx costs in Japan were limited to data from individual university hospitals, which may not accurately reflect the overall cost profile of KTx in Japan. Our study, however, included LDKT and DKT recipients from 11 and 13 distinct institutions, respectively. While our sample may not include all KTx recipients in Japan, its multi-institutional nature provides a more comprehensive and generalizable representation compared to previous single-center studies. Third, only a limited number of factors could be examined in the exploratory analysis because of the absence of laboratory data for patients in this database. Fourth, our study did not include the cost of kidney donation due to the inability of the JMDC database used in this study to link donor and recipient data. Further studies considering both KTx and kidney donation are still warranted. These limitations highlight the need for additional studies to gain a more comprehensive understanding of the factors affecting the cost of KTx. However, despite these limitations, no study has yet reported the cost of KTx in a large general Japanese population. Therefore, this study may be of significant value in evaluating the costs for KTx recipients in Japan.

## Conclusion

This study revealed medical KTx costs in Japan using large medical receipt data. This study enhances our understanding of the effects of KTx on costs and provides valuable information to inform cost-effective and cost–benefit analyses, which aid decision-makers in prioritizing KRT options.

## Supplementary Information

Below is the link to the electronic supplementary material.

Supplementary file1 (PDF 57 KB)

Supplementary file2 (PDF 173 KB)

Supplementary file3 (PDF 62 KB)

Supplementary file4 (PDF 126 KB)

Supplementary file5 (PDF 146 KB)
