# Supplementary material for: The estimation of healthcare cost of kidney transplantation in Japan using large-scale administrative databases
Source: Clin Exp Nephrol. 2024 Nov 20;29(3):350–8. doi: 10.1007/s10157-024-02551-1 (PMC11893673; doi:10.1007/s10157-024-02551-1)
Supplement: Supplementary file 1 — Supplementary file1 (PDF 57 KB) [file 10157_2024_2551_MOESM1_ESM.pdf]

Online Resource 1: The list of procedure codes used in the study

Procedure code	Code name	Purpose of using codes
C102	Home self-peritoneal dialysis management fee	To Identify peritoneal dialysis treatment
J038	Artificial kidney fee	To Identify hemodialysis treatment
K780	Allogeneic cadaveric kidney transplantation procedure	To Identify the implementation of deceased donor kidney transplantation
K780-2	Living donor kidney transplantation procedure	To Identify the implementation of living donor kidney transplantation
