# Supplementary material for: The estimation of healthcare cost of kidney transplantation in Japan using large-scale administrative databases
Source: Clin Exp Nephrol. 2024 Nov 20;29(3):350–8. doi: 10.1007/s10157-024-02551-1 (PMC11893673; doi:10.1007/s10157-024-02551-1)
Supplement: Supplementary file 2 — Supplementary file2 (PDF 173 KB) [file 10157_2024_2551_MOESM2_ESM.pdf]

Online Resource 2

As an exploratory analysis, we examined the factors that contributed to an increase in the costs of LDKT patients as an exploratory analysis. The covariates included patient sex's gender and age at the index date, diabetic kidney disease (DKD), ABO-incompatible KTx, and the use of everolimus. Causes of KFRT, such as DKD and glomerulonephritis, have a significant impact on graft outcomes. We incorporated DKD as a covariates, identified using ICD-10 codes, because it has been reported to be the most common cause of KFRT in KTx recipients in Japan. Patients who underwent ABO-incompatible KTx typically required supplemental procedures, such as rituximab infusion and plasmapheresis, along with an increased use of immunosuppressant drugs after KTx. These additional procedures are thought to result in higher costs than those of ABO-compatible KTx. Therefore, ABO-incompatible KTx was incorporated as a covariates. In this study, ABO-incompatible KTx was defined as the patients who have received rituximab prior to the KTx procedure. Furthermore, we identified the use of four immunosuppressive drugs–: cyclosporine, tacrolimus, mycophenolic mofetil, and everolimus– as their usage patterns of these drugs vary based on the maintenance immunosuppressive regimen of each hospital, and their cost significantly affects the overall cost of post-KTx management. In particular, everolimus is a costly drug, although

the maintenance immunosuppressive regimens including everolimus have been shown to be non-inferior to alternative regimens in terms of the efficacy and safety. Therefore, everolimus was used as the covariates.

We performed a post-hoc exploratory analysis using age as categorical variable as we observed non-linear relationships in the total healthcare costs of both the first year of KTx and subsequent years. We incorporated age as a categorical variable. Generally, costs for younger patients, particularly pediatric recipients, tend to be higher due to more complex surgical procedures and the need for more intensive post-operative care, while costs for older patients may increase due to the management of comorbidities. Given these age-related cost variations, the relationship between age and KTx costs is likely non-linear. Considering these factors, categorizing age into 10-year groups was more appropriate for this analysis.
