# Supplementary figures and images for: The estimation of healthcare cost of kidney transplantation in Japan using large-scale administrative databases
Source: Clin Exp Nephrol. 2024 Nov 20;29(3):350–8. doi: 10.1007/s10157-024-02551-1 (PMC11893673; doi:10.1007/s10157-024-02551-1)

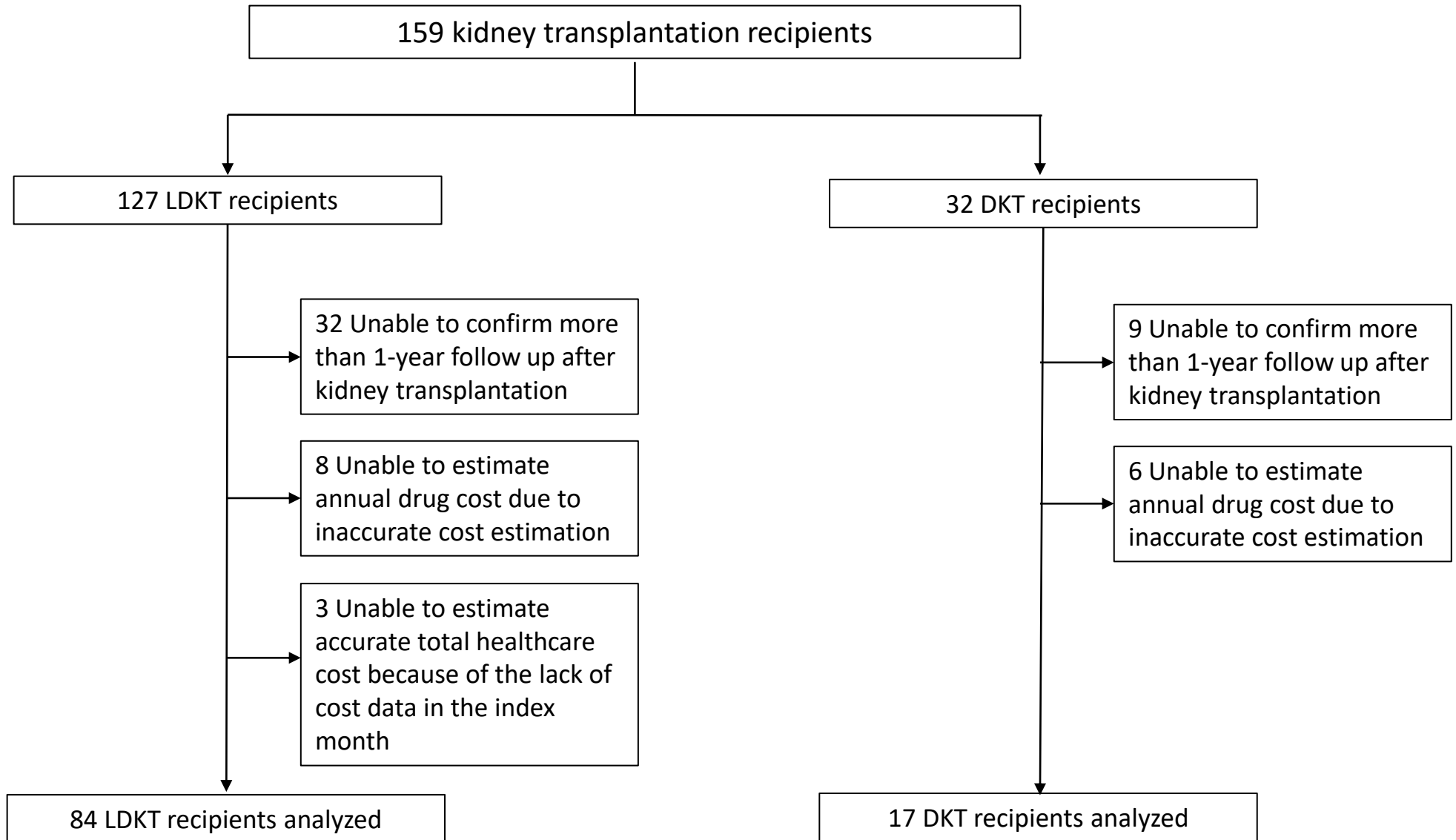

Supplement: Supplementary file 3 — Supplementary file3 (PDF 62 KB) [file 10157_2024_2551_MOESM3_ESM.pdf]
