# Supplementary material for: The estimation of healthcare cost of kidney transplantation in Japan using large-scale administrative databases
Source: Clin Exp Nephrol. 2024 Nov 20;29(3):350–8. doi: 10.1007/s10157-024-02551-1 (PMC11893673; doi:10.1007/s10157-024-02551-1)
Supplement: Supplementary file 4 — Supplementary file4 (PDF 126 KB) [file 10157_2024_2551_MOESM4_ESM.pdf]

**Online Resource 4: Total healthcare costs of living donor kidney transplantation stratified by age groups**

| Age groups | Cost during first year after KTx (JPY)<br>Mean (SD) | Cost after second year post-KTx (JPY)<br>Mean (SD) |
|---|---|---|
| under 29 years | 7,563,799 (404,894) (n=10) | 1,280,086 (201,128) (n=9) |
| 30 to 39 years | 6,648,891 (361,196) (n=16) | 2,390,116 (170,888) (n=16) |
| 40 to 49 years | 6,394,556 (320,796) (n=25) | 1,653,710 (135,842) (n=25) |
| 50 to 59 years | 6,753,500 (323,274) (n=27) | 1,756,121 (184,520) (n=33) |
| over 60 years | 5,588,303 (734,250) (n=6) | 492,145 (112,223) (n=4) |

KTx: Kidney Transplantation; JPY: Japanese Yen; SD: Standard Deviation
