# Supplementary material for: The estimation of healthcare cost of kidney transplantation in Japan using large-scale administrative databases
Source: Clin Exp Nephrol. 2024 Nov 20;29(3):350–8. doi: 10.1007/s10157-024-02551-1 (PMC11893673; doi:10.1007/s10157-024-02551-1)
Supplement: Supplementary file 5 — Supplementary file5 (PDF 146 KB) [file 10157_2024_2551_MOESM5_ESM.pdf]

Online Resource 5: Post-hoc multivariate regression analysis of total healthcare costs of LDKT using age as categorical variant

	First year after living donor KTx*			Subsequent years after living donor KTx †		
	Cost (JPY)	95% CI	P value	Cost (JPY)	95% CI	P value
Male (ref: female)	-101,965	- 824,050 to 620,119	0.78	-144,309	-549,476 to 260,858	0.47
Age category (ref: 40 to 49 years)						
Under 29 years	**1,160,700**	**28,497 to 2,292,902**	**0.045**	-393,816	-1,115,934 to 328,302	0.27
30 to 39 years	469,971	-526,476 to 1,466,418	0.35	**867,559**	**333,867 to 1,401,250**	**0.002***
50 to 59 years	510,592.3	-336,200 to 1,357,384	0.23	81,713	-519,620 to 683,045	0.79
Over 60 years	-1,145,674	-253,171 to 240,359	0.10	**-1,506,226**	**-2,123,248 to -889,205**	**<0.001***
ABO-incompatible KTx (ref: ABO-compatible KTx)	673,673	-166,670 to 1,514,017	0.11	720,132	-82,129 to 1,522,394	0.08
Presence of DKD (ref: absence of DKD)	-160,897	-1,217,525 to 895,732	0.76	-715,563	-1,278,044 to 1,134,932	0.91
Use of everolimus (ref: nonuse of everolimus)	**1,415,486**	**519,630 to 2,311,342**	**0.002***	490,580	-176,930 to 1,158,091	0.15

*Linear regression model

† Linear mixed-effect models: In these models, patients were treated as random-effect variables.

Abbreviations: DKD, diabetic kidney disease, KTx, kidney transplantation; JPY Japanese yen; CI, confidence interval
